# “I’m a Medic” – a web-based, social capital approach to health careers

**DOI:** 10.15694/mep.2018.0000280.1

**Published:** 2018-12-11

**Authors:** Paul Garrud, Gwen Hughes, Sarah Greaves, Shane McCracken, Josh Doyle

**Affiliations:** 1 Division of Medical Sciences and Graduate Entry Medicine; 2 Mangorolla CIC

**Keywords:** Outreach, web-chat, social capital, primary care, widening participation, medicine, health careers

## Abstract

This article was migrated. The article was marked as recommended.

Widening access to medicine in U.K. requires outreach that engages schools in remote areas, schools with below average attainment, and schools serving disadvantaged communities in order to develop a more representative profession and meet serious workforce shortages. The approach reported here embodies ideas about how to develop social and educational capital by facilitating live web chats between school children (13-17 years) and teams of health practitioners. “I’m a Medic” comprised three 2-week events over a 10-month period with circa 900 school students and 22 health professionals from general (family) practices participating. A high proportion (78%) of the students was actively engaged in live chats, asking questions, and voting for the most valuable health practitioner. Questions covered education and training, the nature of the practitioners’ work, political and ethical aspects of healthcare, and a variety of scientific and personal aspects. Evaluation showed a positive increase in career interest and aspiration for science, healthcare and medicine. Teachers would all recommend “I’m a Medic” to colleagues and all bar one would take part again. They reported it was effective in engaging students, improving their confidence in asking questions, and their awareness of general practice and the NHS. Practitioners reported improvements in their understanding of how school students view healthcare professions, their interest in public engagement, and their confidence in communicating their work. Logistic challenges included conflict between scheduled web chats in normal school time and practitioners’ clinical commitments. Nevertheless, the project demonstrated effective engagement across geographic and social/educational barriers, and can provide a valuable mode of outreach, particularly about careers in healthcare.

## Background

The ‘Selecting for Excellence (SfE)’ project ( SEEG, 2014) confirmed the very poor representation of young people from socially and educationally disadvantaged backgrounds in medical school and the medical profession in the UK; indeed, around 80% come from managerial and professional backgrounds. In addition, circa 80% of medical applicants come from only 20% of UK secondary schools and colleges, and roughly half of schools and colleges have no medical applicants at all ( Garrud, 2014). Accordingly, the SfE recommendations included considerable focus on outreach as well as the equity of selection processes and support for widening participation students at university. A subsequent report from the UK Medical Schools Council ( Nursaw, 2016) showed that a substantial number of schools and colleges miss out on engagement with medical schools (indeed, higher education in general): it is likely that there is considerable overlap with those whose students do not apply to medicine.

In addition to this difficulty of access to medical school, nationally there has been growing concern about the shortage of qualified doctors choosing to train in general (family) practice (GP) (e.g. Mathews-King, 2015) against figures that model the need for a 50% increase in GP workforce by 2020 ( RCGP, 2016). In several recent years significant proportions of doctors completing their foundation training (a 2-year post-qualification internship) chose not to apply for specialist training, and those figures suggest we may see critical shortages in other areas than GP as well (e.g. psychiatry, emergency medicine) ( BMA, 2017). In the East Midlands region in 2015 only 69% of GP training places were filled - the lowest in the UK ( HEE, 2015) - prompting particular concern about the long-term viability of primary care in this area. One response to this developing crisis in GP recruitment was a report commissioned by Health Education England (HEE) ( Wass et al, 2016) that identified multiple underlying factors, and recommended a multi-pronged approach to raising awareness of, and promoting positive attitudes towards GP. The present paper reports a widening access project targeting young people in secondary education with the objective or raising awareness and aspiration for careers in primary care.

## Approach

The approach is based on sociological understanding of the barriers to aspiration and participation in medicine. Bourdieu (e.g. Bourdieu, 1986) has emphasized the role of social networks in producing and reproducing inequality; his work suggests that it is worth considering how one may develop the educational and social capital of young people from disadvantaged backgrounds. The approach described here is based on Archer’s work focussing on ‘science capital’ (e.g. Archer et al, 2015) - a concept embodying knowledge, attitudes, networks, and behaviour related to science. In the present case, this is deployed to enhance knowledge about and attitudes towards health careers.

The objectives were:-

• To engage young people from disadvantaged backgrounds and schools with below average levels of attainment;
• To increase aspirations around health careers in primary care, and training as a doctor in particular;
• To develop a positive, informed and realistic view of general practice;
• To engage health professionals working in primary care as ambassadors.

The project used an interactive web-based system - “I’m a Medic, Get me out of here” https://Imamedic.uk - to support live webchats between groups/classes of schoolchildren and a panel of primary care health professionals, with three events from June 2017 to March 2018. This followed the established model of “I’m a Scientist” ( Pontin, 2011). The website also supported asynchronous questions and answers during the 2-week events. Towards the end of each event all the participating children had the opportunity to vote for the practitioner they wanted to win a prize of £500 to spend on further outreach projects.

**Figure 1.**
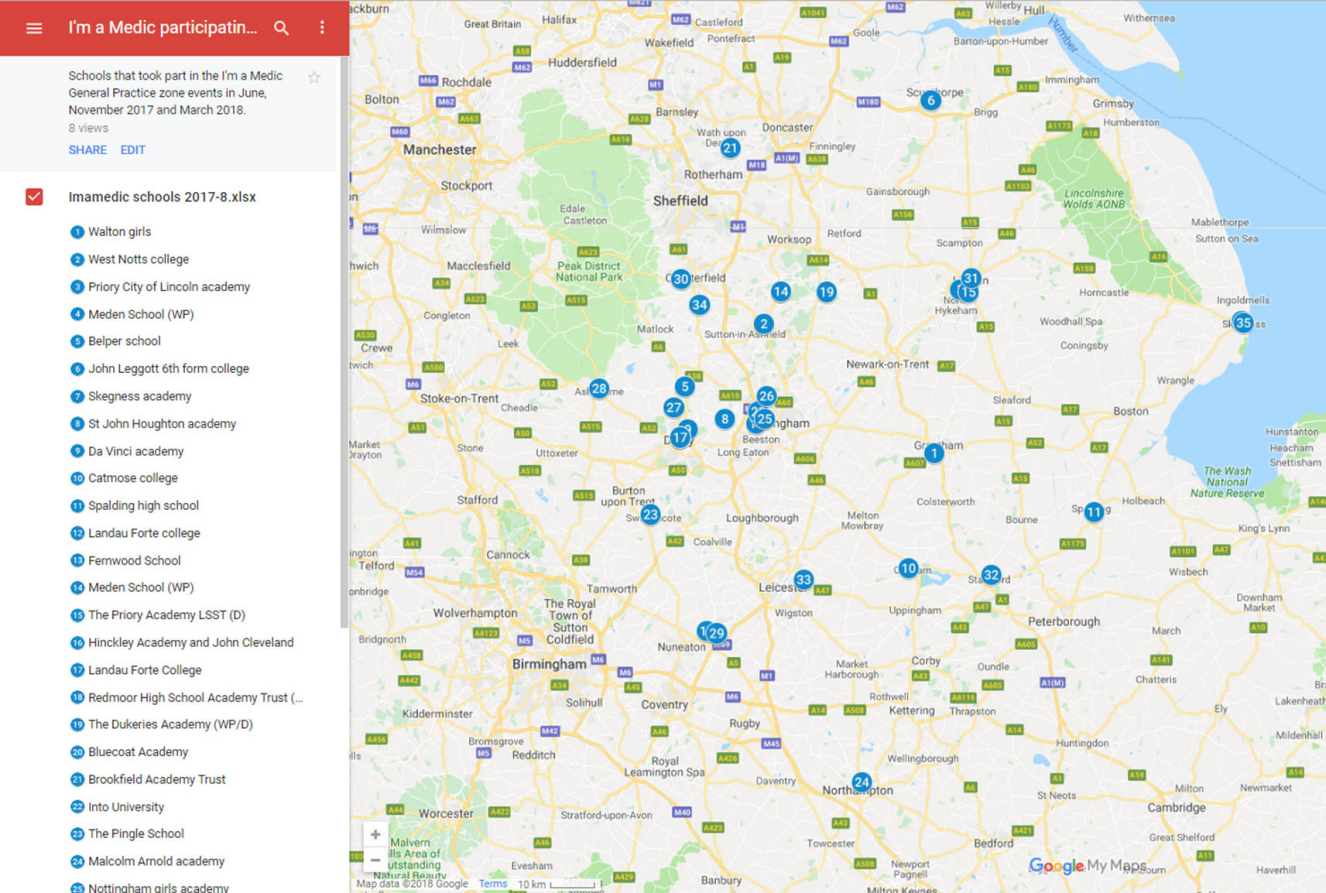
Map of participating schools in East Midlands Map data ©2018 Google

Schools were recruited in East Midlands region via networks from the universities of Nottingham and Leicester widening participation units and Mangorolla, the community interest company who run the “I am a..” websites, using flyers, newsletters, email, etc. The project was also advertised to teachers in the region via existing lists and Twitter. They were invited to book one or more live chats for groups of students in years 9-12 (approx. ages 13-17 years). Teachers were sent a pack comprising suggested preparation and follow-up activities for their students, technical instructions, and a number of careers booklets (e.g. HEE, 2018) and resources (e.g. links to MSC, 2017). In all 34 from a regional target list of 76 schools applied to take part. The distribution can be seen in Figure 1 below.

Healthcare professionals in primary care were recruited from a wide variety of networks including alumni, Local Medical Committees, the Royal College of General Practitioners, and Health Education England: East Midlands, and via social media. A total of seventy practitioners applied to take part and 22 were selected to make up panels of 6, 7 and 8 for the three events in June 2017, November 2017, and March 2018, respectively. They represented a broad range of roles including: qualified GPs, trainee GPs, practice nurses, practice managers, an advanced clinical practitioner and a medical student. Details can be found in the general practice zone reports ( Mangorolla, 2018a, 2018b).

## Impact

Tables 1 and 2 below summarise school and student participation across the three events. They demonstrate that a high proportion of students from the participating schools engaged with the online platform.

**Table 1.** Participation in I’m a Medic events

	Jun 2017	Nov 2017	Mar 2018
Schools	11	12	12
Students logged in	237	272	369
% of students active in ASK, CHAT or VOTE	77%	79%	78%
Questions asked	141	143	169
Questions approved	83	82	109
Answers given	263	239	248
Comments	6	53	30
Votes	100	154	194
Live chats	11	15	16

**Table 2.** I’m a Medic page views

Page views	Jun 2017	Nov 2017	Mar 2018
Total zone	10,229	11,629	10,359
ASK page	501	611	527
CHAT page	1,091	864	879
VOTE page	554	395	516

Just under 900 pupils took part, raising a substantial number of questions that, after moderation to remove duplication, received answers on average from three practitioners. The majority - circa four fifths - of students were active on the site making use of the asynchronous ASK function as well as the live CHATs. Voting for the most useful professional was also popular.

**Figure 2.**
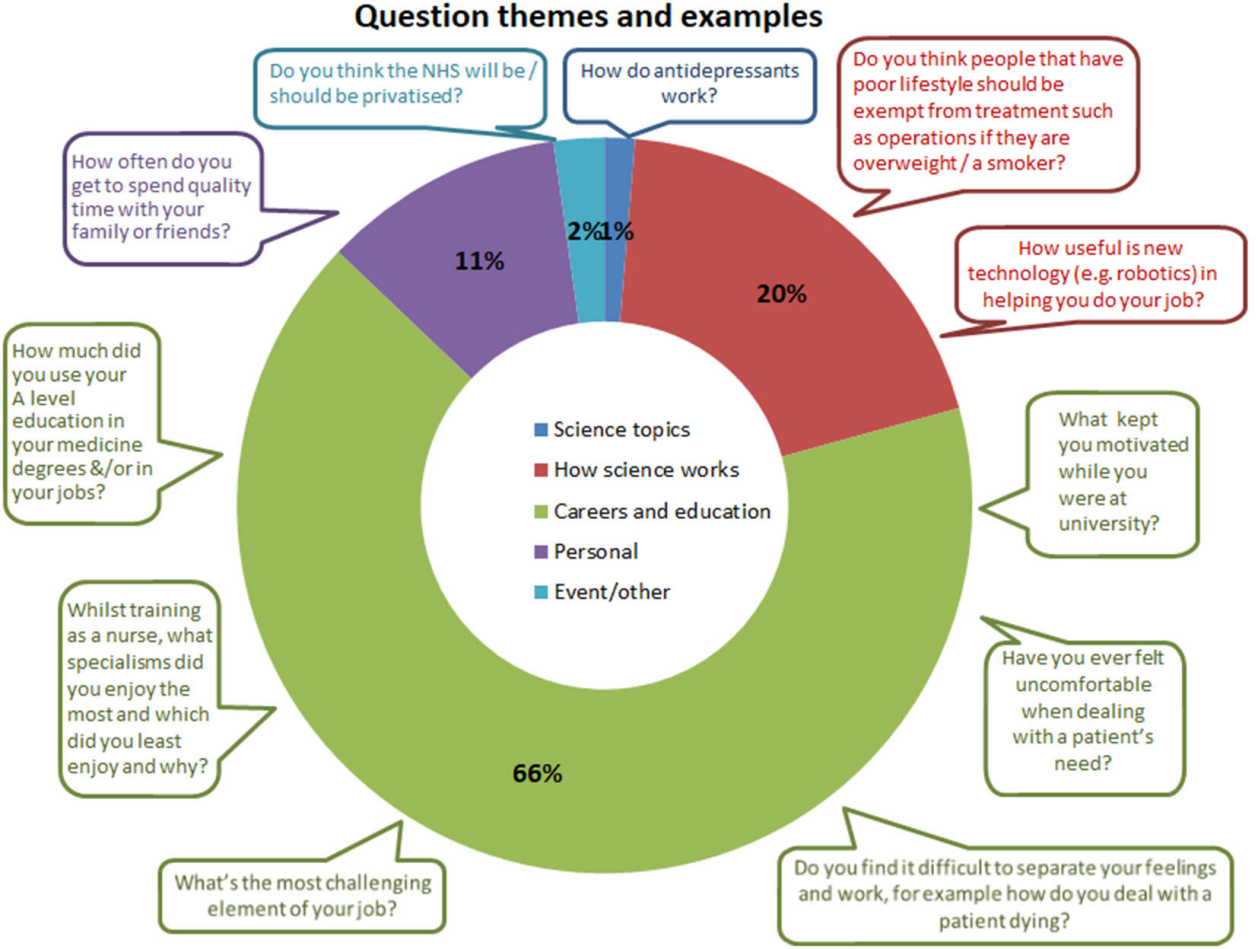
Question types and proportions.

A wide range of questions were asked covering the work of different team members, how they chose to become practitioners, their training, and difficult questions about funding, ethics, health policy and politics, as well as the challenging aspects of working in healthcare. Figure 2 below illustrates the proportions and types of question collated over the 1-year study.

For coding see: https://about.imascientist.org.uk/2017/student-question-coding/

**Figure 3.**
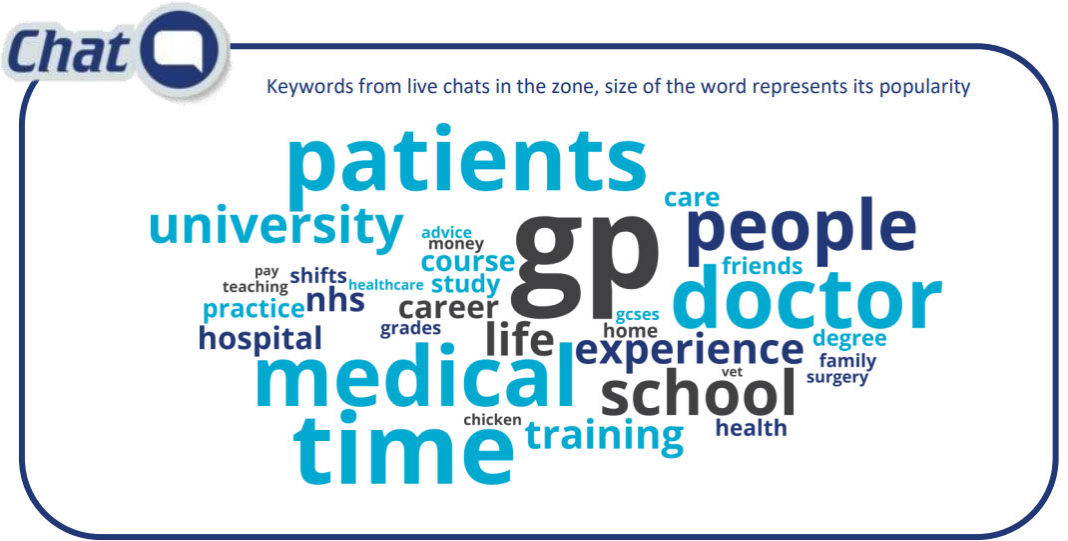
Common terms arising in questions

Figure 3 gives a depiction of the common terms that came up in questions.

## Evaluation

Feedback took multiple forms, including pre- and post-event surveys of participating students, teachers, and health practitioners, and interviews with a purposive sample of teachers and professionals after the conclusion of the third event. Table 3 below summarises the student feedback.

**Table 3.** Student views pre- and post-event

	*For sure / strongly agree*	*I think I probably would / agree*	*Hmmm, ask me in a year... / neutral*	*Not very likely, if I’m honest / disagree*	*Definitely not / strongly disagree*
**When I finish my education, I’d like to have a job that uses my science knowledge and skills**					
Pre	43%	27%	18%	7%	6%
Post	76%	21%	3%	0%	0%
**When I finish my education, I’d like to be a doctor**					
Pre	16%	14%	23%	27%	20%
Post	28%	17%	31%	17%	7%
**When I finish my education, I would like a job in healthcare but I don’t want to be a doctor**					
Pre	18%	23%	26%	20%	13%
Post	24%	24%	29%	16%	7%

As can be seen, there was a positive shift in responses towards jobs using science, being a doctor, and jobs in healthcare (not a doctor).

Teacher feedback on the use of this platform to engage students was very positive, all saying they would recommend “I’m a Medic” to a colleague and all, bar one, that they would take part again; for 85% the project met their expectations entirely or in most aspects. Their views of the impact on student learning is shown in Table 4 below.

**Table 4.** Teacher feedback

How effective do you think I’m a Medic is for the following?					
	*1* *(not at all)*	*2*	*3* *(moderately)*	*4*	*5* *(highly)*
Engaging the students	0.0%	0.0%	15.0%	45.0%	40.0%
Improving students’ confidence in asking questions	0.0%	0.0%	10.0%	55.0%	35.0%
Developing students’ awareness of how the NHS works	0.0%	5.0%	15.0%	45.0%	35.0%
Developing students’ awareness of what it is like to work as a GP	0.0%	0.0%	30.0%	35.0%	35.0%
Developing students’ awareness of different types of healthcare careers	5.0%	10.0%	25.0%	40.0%	20.0%
Developing students’ awareness of the steps needed to follow healthcare careers	5.0%	5.0%	35.0%	30.0%	25.0%

Several features are of note: the success of this approach in engaging students and giving them confidence in asking questions. There was also evidence that they gained awareness of the NHS, about the work of GPs, and about potential careers in healthcare.

Mixed feedback about participating in the venture from the last panel of practitioners is shown in Table 5 below.

**Table 5.** Practitioner feedback

	Strongly decreased	Decreased	Not changed	Increased	Strongly increased
My understanding of how students view healthcare professions has...	0%	0%	33%	33%	33%
My interest in activities where I engage with the public has...	0%	17%	17%	17%	50%
My confidence in communicating my work has...	0%	0%	83%	17%	0%
My skill at communicating my work has...	0%	0%	33%	67%	0%
My enthusiasm towards my own work has...	0%	0%	50%	50%	0%
My appreciation of the value of my work has...	0%	0%	33%	17%	50%

Of the panel, most practitioners reported improvement in their understanding of how school students view them, their interest in public engagement, their skill at communicating their work, their enthusiasm and appreciation of their work and its value, but a proportion still stated little had changed. One practitioner had reduced interest in public engagement afterwards.

## Discussion

The outcomes are discussed below with respect to the initial objectives:-

• *To engage young people from disadvantaged backgrounds and schools with below average levels of attainment;*

The project was successful in engaging a large number of students from secondary schools in East Midlands with lower average levels of attainment; around half of the regional schools targeted took part. This does raise the question of why half of schools did not apply and reasons responsible for a lack of engagement. Logistic constraints may be a contributory factor, but perceived lack of pupil interest or aspiration might also have been influential. In future, the UK government requirements that each secondary school/college meet eight benchmarks in their careers guidance ( Careers & Enterprise Co., 2018) may aid participation in learning about health careers through virtual fora such as “I’m a Medic”.

• *To increase aspirations around health careers in primary care, and training as a doctor in particular;*

There was evidence that following participation in “I’m a Medic”, some students had increased interest in scientific careers in general and were more inclined to consider a career in healthcare as a doctor or in another role, but this was not true for all. Nevertheless, it is arguable that effective careers information should enable young people to see better how well their qualities and interests match those needed in particular jobs and sectors; hence from any school class, one should expect that some gain by realising that, perhaps, this is not for them.

• *To develop a positive, informed and realistic view of general practice;*

Development of a better informed picture of primary care was a clear outcome. To what extent students took away a realistic and/or a positive view was, however, not directly measured. One can assume that practitioners who took part were likely to be more positive about their work and careers than those who didn’t; similarly, professional integrity, may have helped convey a realistic picture. More detailed testing of attitudes after the events may be beneficial to gain confidence about how well this objective was met.

• *To engage health professionals working in primary care as ambassadors.*

Recruitment of health professionals was successful with over 70 applying to take part, and 22 subsequently forming the panels. Their feedback suggested that most would remain engaged (i.e. in “I’m a Medic” or healthcare outreach), and that they gained from their involvement in “I’m a Medic”. One principal issue, however, was the time commitment required to take part in live web-chats. Many were working in full-time roles and found it a challenge to schedule their clinical work to fit with live chats during school hours. As the one-year project proceeded an attempt to accommodate this was made by increasing the number on a panel from 6 to 7 and then to recruit teams of health practitioners - e.g. a group performing a similar role, only one of whom would be expected to contribute to any specific web-chat. Both these manoeuvres helped, but did not entirely solve the problem: as such, this will need to be addressed further in future events if the venture is to be sustainable.

## Conclusion

Outreach in higher education comprises different types of opportunity and modes of engagement. Many require geographic accessibility - a manageable travel time between university and school, for example - to take part in discovery days, master classes, and evening events. Virtual approaches offer one method to overcome that barrier. The particular form used for “I’m a Medic” (“I’m a Scientist”, “I’m an Engineer”, etc.) has a number of further advantages: it facilitates a personal conversation between the professional and the student, it gives control to the student over what is discussed, and it encourages all the students to ask questions, even the shy and less articulate. The results of this project demonstrate that this approach can be effective in engaging large numbers of schools and students in finding out about work and careers in health care.

## Take Home Messages

• Investment in careers outreach risks increasing inequality unless it is designed to be able to reach all schools regardless of geography.
• Online student-led outreach activities allow an equality of opportunity for all students. Traditional face-to-face outreach activities can leave shy and unconfident students less engaged.
• A one year pilot using an interactive web-based system between general practice doctors, healthcare practitioners, and schools successfully raised secondary school pupils’ awareness and aspiration for careers in primary care.
• Participation increased practitioners’ understanding of how pupils view their vocations, their personal appreciation of the value of their work, and interest in participating in public engagement activities.
• Addressing logistical constraints and time commitments practitioners face may be integral to sustaining their engagement with similar outreach ventures.

## Notes On Contributors

Shane’s career traveled through advertising, magazine publishing and broadcast TV before he started a digital engagement company, Gallomanor Communications, in 2001. The company has gone on to produce democratic and STEM engagement projects across the UK and internationally, including I’m a Scientist and I’m a Medic.

Dr Gwen Hughes is an Associate Professor in Physiology, Course Lead for BSc Medical Physiology and Therapeutics and is responsible for pre-clinical physiology in the Graduate Entry Medicine programme. She has long-term involvement in co-ordinating physiology and STEM outreach and widening participation activities mainly for primary school children.

Sarah’s current role is Outreach Development Officer at University of Nottingham, responsible for development and delivery of widening participation projects and partnerships to support fairer access to medicine. She is a professionally qualified youth and community worker. Her previous roles in higher education and the voluntary sector involved leading Inclusion, well being and development projects with vulnerable young people, refugee communities, and International youth development exchange programmes.

PG works as a principal research fellow in the School of Medicine at Nottingham University. He leads a regional widening participation project in East Midlands and chairs the Medical Schools Council Selection Alliance. He is passionate about equitable selection and making sure admission to medical school is evidence-based.

Josh joined the Mangorolla team in 2013 with a background in Chemistry. His responsibilities have included the day-to-day running of the “I’m a..” projects, schools recruitment, and providing teacher support. His current role focuses on evaluation, as well as providing ad hoc event and communications support.

## Declarations

SM is CEO of Mangorolla CIC that operates the “I’m a Scientist”, “I’m an Engineer”, and other outreach and public engagement projects in addition to “I’m a Medic”.JD is employed by Mangorolla CIC.
