# Supplementary material for: “I’m a Medic” – a web-based, social capital approach to health careers
Source: MedEdPublish (2016). 2018 Dec 11;7:280. [Version 1] doi: 10.15694/mep.2018.0000280.1 (PMC10898451; doi:10.15694/mep.2018.0000280.1)
Supplement: Peer review history

# Reviewer report: Reviewer response for version 1

**DOI:** 10.21956/mep.19727.r29547

**Published:** 2019-06-10

**Authors:** Trevor Gibbs

**Affiliations:** 1 AMEE

This review has been migrated. The reviewer awarded 4 stars out of 5 I enjoyed reading this paper and was fascinated by the results. This may reflect my age but I would have thought quite the opposite to the authors in their findings with face-to-face encounters.I wonder also whether the truth was already blurred by this methodology, which I feel is what my co-reviewer was considering. However I do feel that it isa paper worth reading by those engaged with student selection

Reviewer Expertise:

NA

No decision status is available

# Reviewer report: Reviewer response for version 1

**DOI:** 10.21956/mep.19727.r29546

**Published:** 2018-12-14

**Authors:** Richard Hays

**Affiliations:** 1 James Cook University

This review has been migrated. The reviewer awarded 3 stars out of 5 Thank you for the invitation to review this interesting paper. Perhaps it is really two papers intertwined, because it reports two interventions that I think are conceptually different and probably should not be linked for medical school applicants. The strategy to increase interest in medical and health careers among school students is based on substantial evidence from elsewhere and is very worthwhile. The outcome is inline with expectations, and so nothing new, but reports of success in the UK context are welcome. It will be nice to see some follow up of outcomes ove time, to see what impact the strategy has on applications and entry. On the other hand, the attempt to promote GP careers at this level is probably conceptually flawed without attention to addressing the very negative experiences reported later by medical students and GPS. Medical programs can be quite toxic to GP interest - students spend little time there and can be swallowed easily by the lure of high technology, hospital based careers. Then there is the culture of the NHS general practice, which In places has poor morale. While making GP careers a part of the widening participation strategy is OK, the authors should address then broader problems of GP recruitment, or at least refer to this more complex set of issues. That would earn another star.

Reviewer Expertise:

NA

No decision status is available
